# A porcine gluteus medius muscle genome-wide transcriptome analysis: dietary effects of omega-6 and omega-3 fatty acids on biological mechanisms

**DOI:** 10.1186/s12263-017-0552-8

**Published:** 2017-01-31

**Authors:** Magdalena Ogłuszka, Agnieszka Szostak, Marinus F. W. te Pas, Ewa Poławska, Paweł Urbański, Tadeusz Blicharski, Chandra S. Pareek, Edyta Juszczuk-Kubiak, Jenelle R. Dunkelberger, Jarosław O. Horbańczuk, Mariusz Pierzchała

**Affiliations:** 1 Institute of Genetics and Animal Breeding of the Polish Academy of Sciences, Postępu 36A, 05-552 Jastrzębiec, Poland; 2 Breeding and Genomic Centre, Wageningen UR Livestock Research, 6700 AH Wageningen, The Netherlands; 3 Division of Functional Genomics in Biological and Biomedical Research, Centre for Modern Interdisciplinary Technologies, Nicolaus Copernicus University, 87-100 Torun, Poland; 4 Department of Animal Science, Iowa State University, 2255 Kildee Hall, Ames, IA 50011 USA

**Keywords:** Gluteus medius muscle, Omega-3 fatty acid, Omega-6 fatty acid, Pig, Transcriptome

## Abstract

**Background:**

The level of omega-6 and omega-3 polyunsaturated fatty acids can affect many cellular systems and function via nuclear receptors or the bioactive lipid regulation of gene expression. The objective of this study was to investigate changes in the muscle transcriptome and the biological functions regulated by increased consumption of omega-3 and omega-6 fatty acids in the pig gluteus medius muscle.

**Results:**

The transcriptome of the gluteus medius muscle was studied for pigs subjected to either a control diet or a diet supplemented with linseed and rapeseed oil to increase polyunsaturated fatty acid content. Next-generation sequencing (NGS) was used to generate the muscle tissue transcriptome database pointing differentially expressed genes (DEG). Comparative expression analyses identified 749 genes significantly differing at least in the twofold of change between two groups of animals fed with divergent level of omega-3 and omega-6 fatty acids. The expression of 219 genes was upregulated, and the expression of 530 genes was downregulated in the group of pigs supplemented with omega-3 and omega-6 fatty acids in relation to control group pigs. Results of RNA-seq indicated a role of fatty acid in the regulation of the expression of genes which are essential for muscle tissue development and functioning. Functional analysis revealed that the identified genes were important for a number of biological processes including inflammatory response, signaling, lipid metabolism, and homeostasis.

**Conclusions:**

Summarizing, obtained results provide strong evidence that omega-6 and omega-3 fatty acids regulate fundamental metabolic processes in muscle tissue development and functioning.

**Electronic supplementary material:**

The online version of this article (doi:10.1186/s12263-017-0552-8) contains supplementary material, which is available to authorized users.

## Background

The expression of animal genes can be modified in response to environmental factors. One such factor is feed, especially the composition of its bioactive dietary components [1]. The newly emerging field of nutrigenomics investigates the relationship between dietary components and genomic expression profiles [2]. Recently, nutrigenomic research has focused much attention on omega-6 and omega-3 fatty acids.

Omega-6 and omega-3 fatty acids are polyunsaturated fatty acids (PUFA), which belong to the essential fatty acid family and must be obtained from food sources [3]. Mammals can, however, metabolize PUFA by utilizing the omega-6 and omega-3 fatty acid conversion pathways to synthesize other omega fatty acids (i.e., arachidonic acid from linoleic acid (LA), as well as eicosapentaenoic acid and docosahexaenoic acid from alpha-linolenic acid (ALA)) [4]. Omega-6 and omega-3 fatty acids modulate gene expression and provide substrates for the production of signaling molecules or functioning mediators [5]. Additionally, a correct balance of these fatty acids is important for the proper function and development of various cell types [6]. Presence of omega-6 and omega-3 fatty acids and maintaining their optimal ratio in the diet helps to prevent the development of metabolic diseases, such as heart attacks, atherosclerosis, thrombosis, arrhythmia, stroke, immune-inflammatory disorders, asthma, arthritis, cancer proliferation, type II diabetes mellitus, obesity, and psychiatric disorders [7]. Although PUFA are known to have significant biological roles, their mechanisms are not well understood.

Fatty acids are an important cellular component and serve as a source of energy for animals [8]. A major site of fatty acid catabolism in mammals is the skeletal muscle [9]. In fact, it has been suggested that PUFA content can modulate both the function and metabolism of the skeletal muscle. The effect of PUFA on the skeletal muscle also has the potential to substantially impact whole body functioning [10]. PUFA content is important for muscle tissue as well, regulating immunological, hormonal, and metabolic processes [11]. Gluteus medius muscle was selected as the target muscle tissue for this study due to its dynamic energy metabolism and role in independent gait [12]. The gluteus medius muscle contains IIb and IIx fibers as well as IIa (fast oxidative-glycolytic) fibers [13], which can switch between glycolytic and oxidative metabolism in reaction to accessibility of the fatty acids [14]. Although the immune-stimulating effects of PUFA have been studied for decades [15], knowledge of their integrative effects is limited. Therefore, the objective of this study was to investigate changes in the pig gluteus medius muscle transcriptome, including the regulated biological functions as a result of increased omega-3 and omega-6 fatty acid concentration. In our studies, we used pigs, which are one of the best models for studies on effects of omega-6 and omega-3 fatty acids supplementation. Moreover, they are a proven and recognized large animal model due to their anatomical and physiological similarities to humans. We hypothesize that supplementation of omega-6 and omega-3 fatty acids in the diet will modulate the expression of genes essential for muscle homeostasis.

## Methods

### Animals

Ethical clearance was obtained from the Local Ethics Commission for Experimentation on Animals in Warsaw (No 27/2009). Analyses were conducted on Polish Landrace. The breed was based on improved white German pig and was upgraded using German and Swedish national breeds. Unrelated female pigs (*n* = 24) were bred at a commercial farm and fed with a standard diet until pigs reached approximately 60 kg in body weight (105 ± 2 days of age). Pigs were then divided into two groups and fed with one of two diets until slaughter. Pigs fed with the control diet received a regular feed mixture (Table 1) of 268 mg of LA and 25 mg of ALA/100 g. Pigs fed with the experimental (supplemented) diet received additives of 1% rapeseed oil and 2% linseed oil to the standard diet in the form of 660 mg of LA and 64 mg of ALA/100 g. The fodder mixtures (identical to the fodders used in Szostak et al. [16]) were isocaloric (13 MJ EM per kg of mixture) and balanced according to the amino acid composition. Pigs were delivered to a commercial slaughterhouse at least 24 h prior to slaughter once they reached a body weight of approximately 110 kg (168 ± 2 days of age). Pigs were sacrificed by exsanguination after electrical stunning, reflecting industry standards. Gluteus medius muscle samples were taken immediately after slaughter, frozen in liquid nitrogen, and stored at −80 °C for further analysis.

**Table 1:** Composition of pigs diets

	Experimental diet	Control diet
Ingredient %		
Rapeseed meal	7.2	6.0
Soybean meal	9.2	9.0
Wheat	49.6	54.0
Barley	28.5	28.5
Linseed oil	2.0	0
Rapeseed oil	1.0	0
Other	2.5	2.5

### Gas chromatography determination of the ratio of omega-6 to omega-3 fatty acid

Quantitative data for the ratio of omega-6 to omega-3 fatty acid in all gluteus medius muscle samples were obtained by gas chromatography using flame ionization detection (GC–FID) analysis. For each sample, 1 g of muscle tissue was homogenized and extracted with chloroform-methanol 2:1(*v*/*v*) according to Folch et al. [17]. Fatty acids were analyzed using a GC-7890 Agilent gas chromatograph (Agilent Technologies, Inc., Santa Clara, CA,) equipped with a 60-m capillary column (Hewlett-Packard-88, Agilent J&W GC Columns, Santa Clara, CA) with 0.25-mm inner diameter and coating thickness of 0.20 μm. Helium was used as a carrier gas at a flow rate of 50 mL min^−1^. The injector and detector were both maintained at 260 °C. Column oven temperature was programmed to increase from 140 °C (held for 5 min) at a rate of 4 °C min^−1^ to 190 °C to 215 °C at a rate of 0.8 °C min^−1^. Individual fatty acid peaks were identified by comparison with known reference methyl esters (Supelco 37 Component FAME Mix, 47885-U, Sigma-Aldrich Co. Warsaw, Poland) and expressed as a percentage of total fatty acid concentration [18].

### RNA isolation

To prepare samples for next-generation sequencing [19], 20 mg of frozen tissue were pulverized with a mortar and pestle precooled with liquid nitrogen. The RNA was isolated using an mRNA extraction kit (RNeasy Fibrous Tissue Mini Kit—Qiagen, Crawley, UK) according to the manufacturer’s recommendations. For real-time PCR, 20 mg of frozen tissue was homogenized in 800 μl of TRIzol reagent (Invitrogen, Carlsbad, CA) using tubes with ceramic beads on Magna Lyser (Roche Applied Science, Penzberg, Germany). The extracts of total cellular RNA were isolated from homogenate according to Chomczynski and Sacchi [20]. The concentration and purity of total RNA were checked using a UV-Vis spectrophotometer (Actgene, Piscataway, NJ) at 260 nm. The integrity of ribosomal RNA was evaluated electrophoretically on the Bioanalyser (Agilent Technologies Inc.). Only high-quality RNA isolates, characterized by an RNA integrity number greater than 7, were used for further analysis.

### Next-generation sequencing

Equal amounts of total RNA of six randomly selected individuals per group were used to construct libraries for sequencing. The mRNA-Seq library was constructed and sequenced by Genomed (http://www.genomed.pl/, Poland) [21] in accordance with the manufacturer’s instructions for the Illumina MiSeq2000 system (Illumina, Inc., San Diego, CA, USA). The TruSeq RNA Sample Pre Kit (Illumina Inc.) was used to construct the libraries. To remove rRNA, bead-based depletion was used. Libraries were synthetized twice from freshly prepared RNA pools (each pool contained material from six pigs) and were sequenced independently on the MiSeq high-throughput sequencing instrument with 150 paired-end sequencing. Obtained sequence reads were aligned to the Sus scrofa 10.2 genome (NCBI GenBank ID GCA_000003025.4). Preprocessing and mapping was performed using the CLC Genomics workbench (CLC bio, Denmark) and DNASTAR Lasergene Genomic Suite (DNASTAR, Inc., Madison, WI). The read mapping was conducted using the RNA-Seq analysis package default settings (mismatch cost 2, insertion cost 3, length fraction 0.8, similarity fraction 0.8, and maximum number of reads 1) within the CLC software. The number of mapped reads was normalized and measured in terms of reads per kilobase per million (RPKM) to determine transcript abundance. For this study, pooled sample was used in order to remove animal-specific differences, allowing for the general reaction mechanism to be highlighted (for comparison, see te Pas et al. [22] and Szostak et al. [16]). As there was a single sample comprised of pooled individuals, in each condition, genes with the highest expression variance were estimated by using the variance across the two conditions. This is known as a “blind” method of dispersion estimate. The analysis assumes that for most genes, there is no true differential expression and that a valid mean-variance relationship can be estimated from treating the two samples as if they were replicates what will lead to emergence of a minority of differentially abundant genes acting as outliers. This method results in overly strict statistical significance testing of differential gene expression [23]. Genes that showed at least a twofold change in expression between control and experimental group and had a *t* test *P* value ≤0.05 adjusted for false discovery rate (FDR) were considered for detailed functionally grouped network analysis using DAVID software and Cytoscape 3.2 ClueGO plugin.

### Reverse transcription PCR

DNase digestion was performed with RQ1 RNase-free DNase (Promega Corp., Madison, WI) by incubating samples with DNase for 15 min at 20 °C. One microgram of total RNA was reverse transcribed into cDNA using oligo-dT primers and the Transcriptor First Strand cDNA Synthesis Kit (Roche Applied Science) following the manufacturer’s recommendations.

### Real-time PCR

To validate the NGS data, the expression level of 10 genes was analyzed using two-step quantitative real-time PCR. More pigs were used for validation and were subjected to the same conditions as those involved in NGS analysis: 12 pigs were fed with the experimental fodder and 12 pigs were fed with the control fodder. All samples were analyzed individually. Selected genes were differentially expressed between the two diet treatments. Expression was normalized to the topoisomerase (DNA) II beta (*TOP2B*) gene as an endogenous control, and intron-spanning primers were specifically designed to quantify target mRNA transcripts. Genes and their primers are presented in Table 2. All reactions were performed on the Light Cycler 480 (Roche Applied Science) using the SYBR Green methodology. The standard curve to determine efficiency of the real-time PCR reaction and the appropriate dilution of cDNA template were determined based on a serial dilution of pooled cDNA samples. Cycling conditions were as follows: 95 °C for 5 min followed by 50 cycles (95 °C for 10 s, 58 °C for 10 s, and 72 °C for 10 s). A melting curve was performed from 60 to 95 °C to check the specificity of the amplified product. Real-time PCR was achieved on RNA samples from individual animals. Gene expression levels were calculated using the linear regression method for analyzing real-time PCR data [24]. To determine relative genes expression for independent samples, the bilateral Student *t* test was used; *P* value ˂0.05 was considered as significant.

**Table 2:** Real-time PCR primer

Gene symbol	Description	Forward primer	Reverse primer	Product length (bp)
TTR	Transthyretin	CTACTGGAAGGCACTTGGCA	AGGGCTGTGGTGGAGTAAGA	132
APOC3	Apolipoprotein C-III	AGGACACCTCCCTTCTGGAC	GTCGGTGAACTTGCCCTTGA	179
RBP4	Retinol-binding protein 4	CGGACTATGACACCTACGCC	GTGACACCCTCCATGTTGCT	245
ADAM19	ADAM metallopeptidase domain 19	AGTTTACGAACCAGACCAAGAA	GCATAATCAGCCACAAGGTAAAG	97
FADS1	Fatty acid desaturase 1	CGTGATTGACCGGAAGGTGT	TAGTCGGTTCAAAGCTGGGC	203
FADS2	Fatty acid desaturase 2	GCGCAGATGCCTACCTTT	TAAATCAAGGTCGCGGTGGAA	198
ADORA1	Adenosine A1 receptor	CCTCTTCCTCTTTGCCCTTAC	GTAGATGAGGATGCTGGACTTG	97
CASQ1	Calsequestrin 1	CCAACAGCGAAGAGGAGATT	TCCTCCCAGGTCTCATACATAC	97
MAPK1	Mitogen-activated protein kinase 1	CCCTCACAAGAGGATTGAAGTAG	GGCTCATCACTTGGGTCATAA	78
FADD	Fas (TNFRSF6)-associated via death domain	GGGCGGGAAGTGTTTGATT	CTCCCTGGCCAATTCTGTTATG	83
CCL4	C-C motif chemokine ligand 4	GCAAGACCATGAAGCTCTGC	AAGCTTCCGCACGGTGTATG	137
TOP2B	Topoisomerase (DNA) II beta	AACTGGATGATGCTAATGATGCT	TGGAAAAACTCCGTATCTGTCTC	464

### Functional bioinformatics analysis

#### DAVID analysis

Differentially expressed genes (DEG) that showed at least a twofold change in expression between control and experimental group and had a *t* test *P* value ≤0.05 adjusted for FDR were selected for DAVID analysis (The Database for Annotation, Visualization, and Integrated Discovery: https://david.ncifcrf.gov/ [25]). DAVID software calculates *P* values for the enrichment of the number of genes in biological mechanisms. These *P* values indicate the importance of the general biological mechanisms active in all animals but do not indicate the inter-individual differences. All DEG were analyzed. The gene list was transferred to NCBI’s ENTREZ search engine of cross-database, which recognized 493 unique gene names. Of these 493 genes, 481 of the gene IDs were transferred to human gene IDs, to compensate for poor pig gene annotation. Further analyses were performed using the total human genome as a background. An error rate of 0.05 was assumed significant according to the Benjamini and Hochberg multiple test correction method, and a FDR less than 5% was applied.

#### Cytoscape analysis

To further elucidate the biological relevance of the DEG, a functional enrichment analysis was performed using ClueGO software, a Cytoscape 3.1.0 plug-in (Institute for Genomics and Bioinformatics, Graz University of Technology, Graz, Austria) [26]. ClueGO facilitates the visualization of functionally related genes by displaying the genes as a clustered network and chart. The statistical test used to determine the enrichment score for terms and groups was based on a right-sided hypergeometric distribution option with a Benjamini-Hochberg correction. A kappa statistic was calculated to determine the strength of the connection between the terms in the network, based on similarity of associated genes [26]. To obtain complex, clear, and readable networks, various kappa scores were applied. The kappa score was preset to 0.9 for analyses covering a complete list of genes. A minimum level of 7 and a maximum level of 15 were set as the GO level interval with a minimum of two genes per category.

## Results

### Phenotype parameters

There were no significant differences in carcass nor meat quality traits between pigs of the two dietary groups. For animals from the experimental group, we observed a tendency in the reduction of backfat thickness and fodder intake.

The effect of dietary treatment on fatty acid composition in pig gluteus medius muscle is presented in Table 3. The greatest difference between diets was detected for the omega-3 fatty acid level, for which the mean value of the supplemented diet was more than five times that of the control diet (*P* value <0.001). In contrast, there was no evidence of a difference in omega-6 fatty acid level in the muscle of pigs fed with different diets (*P* value >0.05). Pigs fed with the supplemented diet had significantly higher levels of polyunsaturated fatty acids (*P* value <0.05) and decreased levels of monounsaturated fatty acids (*P* value <0.05) compared to pigs fed with the control diet.

**Table 3:** Fatty acid profile [g/100 g FAME] of the muscle of pigs from two examined groups (mean ± SD)

Fatty acid	Supplemented	Control	*P* value
C14:0	1.39 ± 0.18	1.51 ± 0.32	0.42997
C16:0	23.77 ± 1.35	25.17 ± 1.67	0.13779
C18:0	13.96 ± 0.54	13.75 ± 0.57	0.84922
C20:0	0.27 ± 0.13	0.11 ± 0.07	0.66062
C16:1 n-7	2.78 ± 0.34	3.34 ± 0.42	0.02783*
C18:1 n-9	36.23 ± 1.93	38.92 ± 2.02	0.04020*
C18:1 n-7	3.34 ± 0.32	3.92 ± 0.31	0.01022*
C18:2 n-6	9.44 ± 1.49	8.14 ± 2.46	0.29516
C18:3 n-3	4.45 ± 1.09	0.57 ± 0.52	1.37E−5***
C20:4 n-6	1.24 ± 0.24	1.36 ± 0.53	0.62428
Ʃ saturated FA	41.85 ± 1.30	42.87 ± 1.46	0.23132
Ʃ monounsaturated FA	42.77 ± 2.41	46.48 ± 2.54	0.02678*
Ʃ polyunsaturated FA	15.27 ± 2.66	10.19 ± 2.20	0.00476**
Ʃ n-6 FA	10.69 ± 1.67	9.51 ± 2.79	0.39499
Ʃ n-3 FA	4.47 ± 1.14	0.89 ± 0.55	0.00035***
n-6/n-3 FA	2.57 ± 1.09	11.57 ± 8.81	0.120

Means and standard deviations of fatty acids and profiles of omega-6/omega-3 fatty acids. Comparisons were made between the muscle tissue of pigs fed with a diet supplemented with fatty acids versus pigs fed with a control diet

*P value <0.05; **P value <0.01;*P value <0.001

### Next-generation sequencing

NGS was performed to identify changes induced by omega-6 and omega-3 supplementation in the pig muscle transcriptome. The number of reads per sample obtained from NGS was 25,244,670. Among these reads, 16,040,530 (63.54%) were mapped in pairs and 792,066 (3.14%) in broken pairs and 8,412,074 (33.32%) were unmapped. The number of counted fragments was 8,020,265 (63.54%); among these fragments, 7,475,220 (59.22%) were unique and 545,04 (4,32%) were not specific. The number of uncounted fragments was 4,602,070 (36.46%). The sequence reads have been submitted to the NCBI Gene Expression Omnibus under accession series GSE83729. The expression of 14246 genes was registered in the muscle tissue of pigs subjected to NGS. An Additional file 1 shows the heat maps of the transcription levels between control and experimental group. Between genes complying the condition of significance (at least a twofold change in expression between control and experimental group and a *t* test *P* value ≤0.05 adjusted for FDR), expression of 219 genes was upregulated and the expression of 530 genes was downregulated.

### Real-time PCR verification of NGS data

**Fig. 1:**
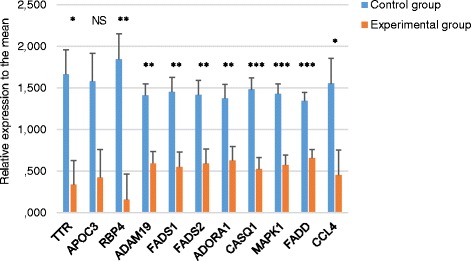
The validation of NGS results with real-time PCR—comparison of control and experimental group. Mean expression of ten genes: transthyretin (*TTR*), apolipoprotein C-III (*APOC3*), retinol-binding protein 4 (*RBP4*), ADAM metallopeptidase domain 19 (*ADAM19*), fatty acid desaturase 1 (*FADS1*), fatty acid desaturase 2 (*FADS2*), adenosine A1 receptor (*ADORA1*), calsequestrin 1 (*CASQ1*), mitogen-activated protein kinase 1 (*MAPK1*), Fas (*TNFRSF6*)-associated via death domain (*FADD*), and C-C motif chemokine ligand 4 (*CCL4*) determined using real-time PCR for 24 pigs from two diet treatments. Expression was normalized to the topoisomerase (DNA) II beta (TOP2B) gene as an endogenous control. *Error bars* represent standard error.*P value <0.05; **P value <0.01; ***P value <0.001, *ns* not significant

**Fig. 2:**
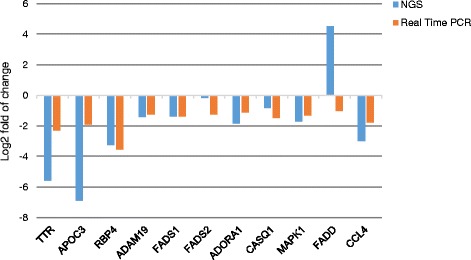
The validation of NGS results with real-time PCR—comparison of two methods. Log2 fold changes of ten differentially expressed genes measured by NGS (*blue*) versus real-time PCR (*orange*): transthyretin (*TTR*), apolipoprotein C-III (*APOC3*), retinol-binding protein 4 (*RBP4*), ADAM metallopeptidase domain 19 (*ADAM19*), fatty acid desaturase 1 (*FADS1*), fatty acid desaturase 2 (*FADS2*), adenosine A1 receptor (*ADORA1*), calsequestrin 1 (*CASQ1*), mitogen-activated protein kinase 1 (*MAPK1*), Fas (*TNFRSF6*)-associated via death domain (*FADD*), and C-C motif chemokine ligand 4 (*CCL4*)

The reliability of differentially expressed genes detected by NGS was confirmed by real-time PCR analysis for ten selected genes. Results are presented in Figs. 1 and 2. Nine of ten genes were significantly differentially expressed in examined groups. For nine out of ten genes, the direction of change identified by real-time PCR was the same as that detected by NGS.

### Functional analysis by DAVID

The DEG in pigs fed with alternative diets were analyzed by DAVID functional clustering analysis. Results identified more than 15 functional clusters (Table 4, Additional file 2). The cluster with the highest enrichment score was related to response to wounding/inflammation. Related clusters included complement and coagulation cascades, acute inflammatory response, and inflammatory response. Decreased expression levels were observed in several genes encoding the chemokines: C-C motif chemokine ligand 2 (*CCL2*), C-C motif chemokine ligand 4 (*CCL4*), C-C motif chemokine ligand 5 (*CCL5*), C-C motif chemokine ligand 16 (*CCL16*), C-C motif chemokine ligand 19 (*CCL19*), and C-C motif chemokine ligand 21 (*CCL21*). Moreover, the expression of complement components (complement component 4 binding protein alpha (C4BPA), complement C5 (C5), complement C7 (C7), complement C8 gamma chain (C8G), and complement C9 (C9)) were observed as downregulated. Finally, we registered changes in the level of two coagulation factors—coagulation factor II, thrombin (F2), and coagulation factor V (F5). Other clusters included processes related to response to endogenous stimulus and organic substance, regulation of response to external stimulus, and negative regulation of response to stimulus. Genes with significantly differential expression were related to retinol and lipid binding, as well as gluconeogenesis, monooxygenase, and microsome. Among the genes related to lipid metabolism, supplementation occurred to decrease expression number of genes coding apolipoproteins, apolipoprotein A1 (*APOA1*), apolipoprotein A2 (*APOA2*), apolipoprotein A4 (*APOA4*), apolipoprotein A5 (*APOA5*), apolipoprotein C3 (*APOC3*), apolipoprotein E (*APOE*), and apolipoprotein N (*APON*). Obtained results of NGS data at the transcriptomic level indicated that omega-6 and omega-3 fatty acid level affected biological processes responsible for keeping homeostasis in muscle tissue.

**Table 4:** Results of DAVID functional clustering analysis of differentially expressed genes (DEG)

Functional annotation	Enrichment score	Number of DEG	Ontology
Response to wounding/inflammation	7.34	48	Biological processes
Signal/extracellular region	5.32	157	Molecular function
Response to endogenous stimulus and organic substance	4.40	43	Biological processes
Regulation of response to external stimulus	3.70	20	Biological processes
Homeostasis	3.30	44	Biological processes
Complement and coagulation cascades	2.43	19	Biological processes
Retinol binding/vitamin A	2.20	7	Molecular function
Oxidoreductase	2.17	30	Molecular function
Lipid binding	1.90	56	Molecular function
Negative regulation of response to stimulus	1.85	11	Biological processes
Gluconeogenesis	1.83	25	Biological processes
Acute inflammatory response	1.81	38	Biological processes
Inflammatory response	1.77	28	Biological processes
Monooxygenase	1.75	10	Molecular function
Microsome	1.52	10	Cellular component

Analysis of genes identified with differential expression in the muscle of pigs fed with a diet supplemented with fatty acids versus pigs fed a control diet

### Functional analysis by Cytoscape

**Fig. 3:**
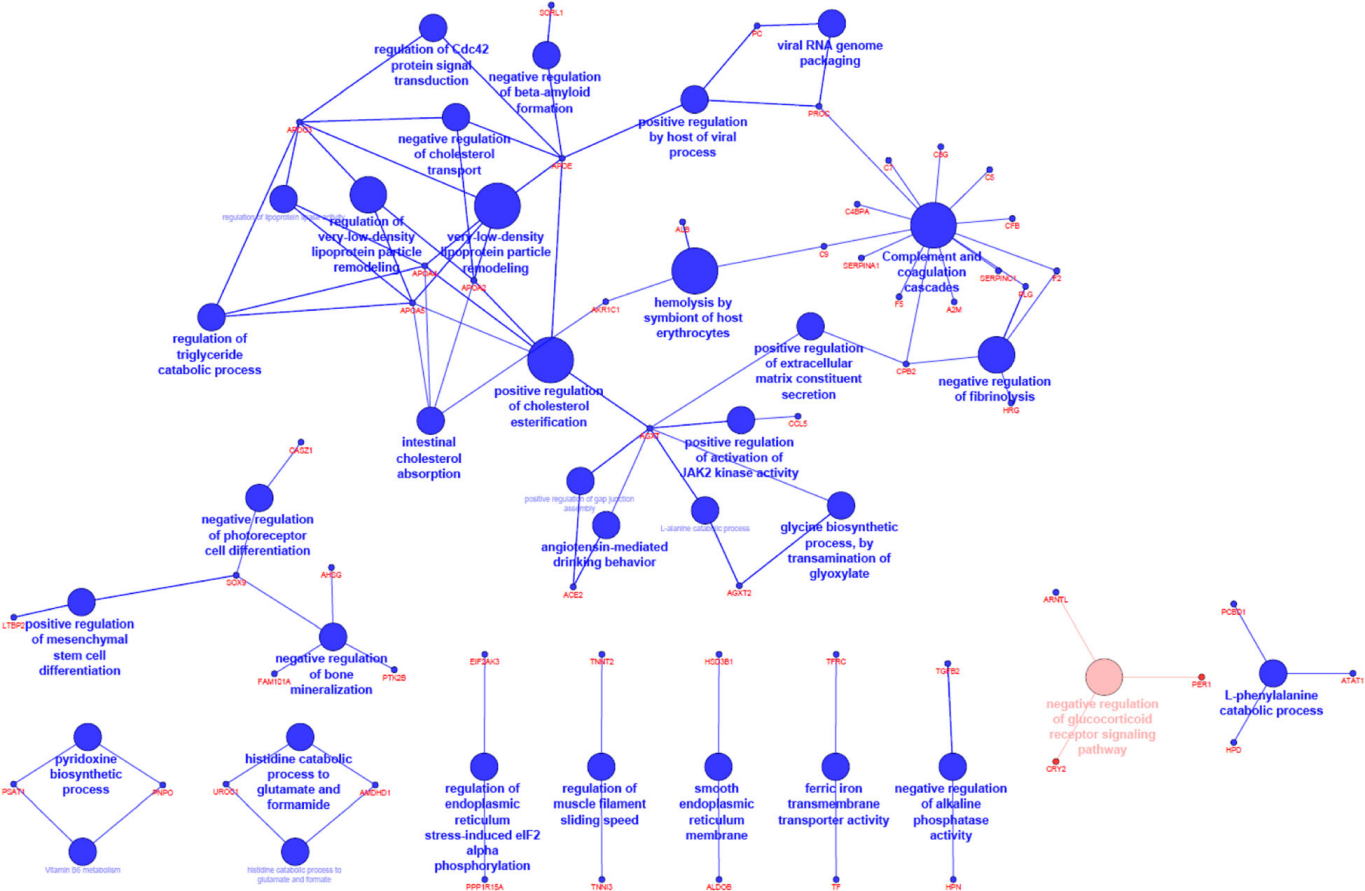
Cytoscape-ClueGo analysis of differentially expressed genes (DEG). Gene networks of upregulated (*red*) and downregulated (*blue*) genes for the diet supplemented with n-6 and n-3 fatty acids versus the control diet. The edges connecting the nodes are based on the kappa statistic (kappa score of 0.9) that measures the overlap of shared genes between terms

**Fig. 4:**
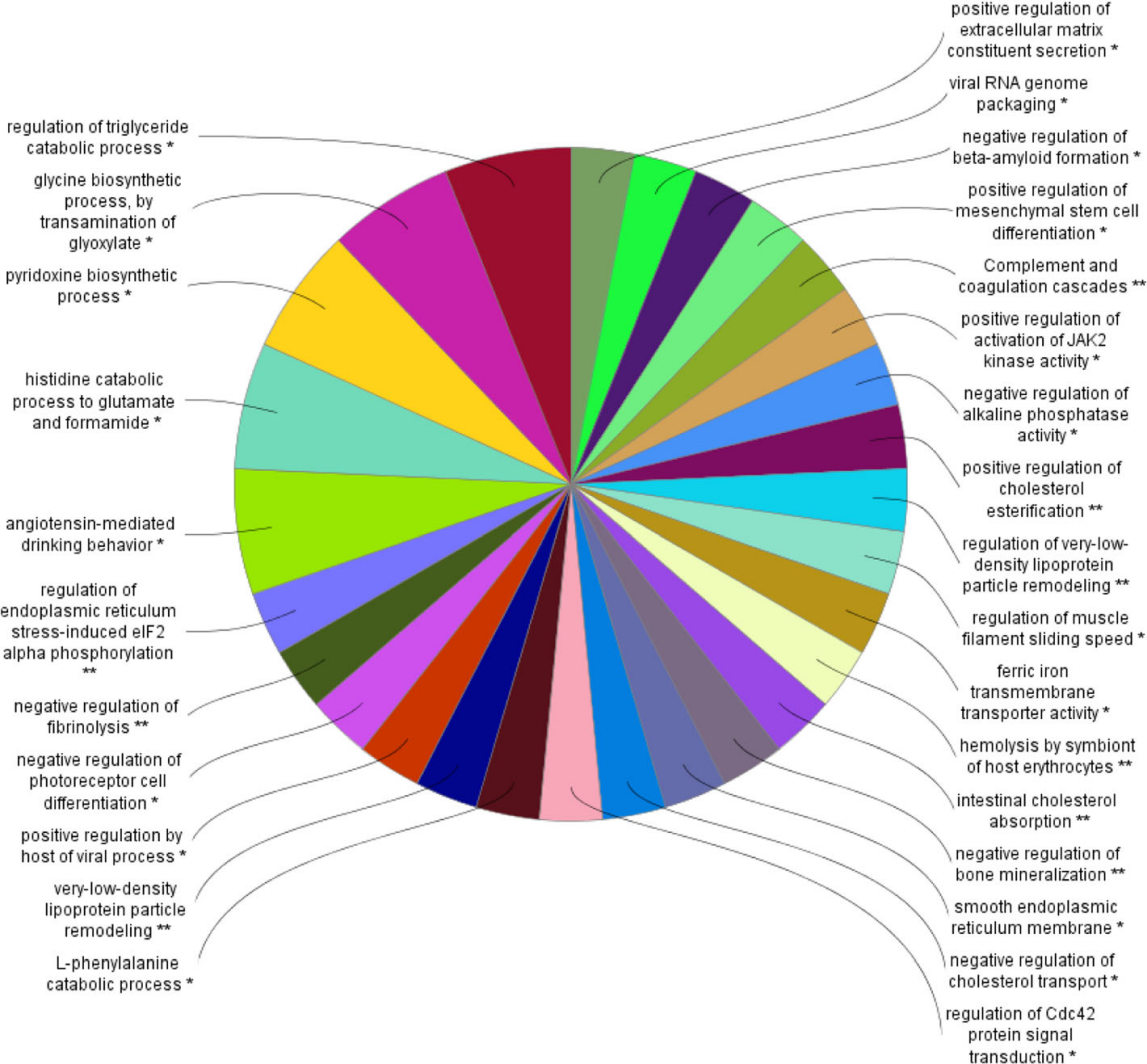
Cytoscape-ClueGo analysis of functional distribution of differentially expressed genes (DEG). Analysis of genes identified with differential expression in muscle of pigs fed with a diet supplemented with fatty acids versus pigs fed a control diet. Categories of genes were based on GO annotation. Significant terms (Kappa = 0.9) and distribution were determined according to percentage of number of gene association

To gain insight into the network interactions of DEG in the muscle tissue of pigs fed with the supplemented diet compared to pigs fed with the control diet, we performed a clustered functional analysis using the Cytoscape software with the ClueGO plug-in. Overall, 34 GO terms were significantly enriched (Figs. 3 and 4, Additional file 3). Among various biological processes, the most affected revealed by functional network analysis were as follows: complement and coagulation cascades, very low-density lipoprotein (VLDL) particle remodeling, negative regulation of fibrinolysis, positive regulation of cholesterol esterification, and intestinal cholesterol absorption (Fig. 3). Several of highlighted terms were related to the same processes: regulation of triglyceride catabolic process and regulation of lipoprotein lipase activity, angiotensin-mediated drinking behavior and positive regulation of gap junction assembly, and vitamin B6 metabolism and pyridoxine biosynthetic process.

## Discussion

The objective of this study was to determine the impact of omega-6 and omega-3 fatty acid concentration on the pig muscle transcriptome. Results provide new insight regarding the possible underlying regulatory mechanisms of the muscle tissue’s biological response to increased concentration of omega-6 and omega-3 fatty acids.

RNA-seq of muscle tissue identified 759 genes with at least a twofold change in gene expression. The number of downregulated genes in pigs characterized by decreased omega-6 to omega-3 ratio in the muscle (for the supplemented diet) was more than two times greater than the number of upregulated genes in pigs fed with the control diet. Duan et al. [6] reported a dietary ratio of omega-6/omega-3 fatty acids of 1:1 and 5:1, which suppressed gene expression levels of lipid metabolism-related genes and proteins, as well as inflammatory cytokines. The authors concluded that their reported ratio of omega-6 to omega-3 fatty acids is beneficial for lipid metabolism and function of the inflammatory system. However, until now, a few studies have investigated the effect of fatty acid consumption on the muscle transcriptome using high-throughput technology.

Omega-6 and omega-3 fatty acids regulate the inflammatory response by nuclear receptors, which regulate gene expression [27] and synthesize pro- and anti-inflammatory biomarkers. Indeed, the most affected process pointed out both by DAVID and Cytoscape-ClueGo was the response to wounding and inflammation. We observed decreased expression of a number of chemokines: *CCL2*, *CCL4*, *CCL5*, *CCL16*, *CCL19*, and *CCL21*, recruiting macrophages and monocytes to the site of inflammation. That may indicate a diminished activity of macrophages and monocytes themselves. Similar results were obtained by Liang et al. [28], who registered decreased expression of *CCL2* after the administration of omega-3 fatty acid to mice. Further, we observed diminished expression of several complement components, *C4BPA*, *C5*, *C7*, *C8G*, and *C9*, that are components of the complement system, a part of the innate immune system. The usefulness of polyunsaturated fatty acid immunomodulatory activities is well known. Their possible impact on a group of chemokines and complement components explore mechanisms of its acting. That supports their utility in the management of inflammatory and autoimmune diseases.

Among genes with divergent expression that are involved in coagulation, two coagulation factors—*F2* and *F5—*showed reduced expression. These genes encode essential enzymes of the blood coagulation cascade. There are a number of studies on the impact of polyunsaturated fatty acids on coagulation process; however, their results are inconsistent. It is suggested that increased consumption of polyunsaturated fatty acids decreased the blood levels of several coagulation factors and may prevent thrombosis [29–31]. Here, we suggest that the possible mechanism of this phenomenon could be related to diminished levels of *F2* and *F5* due to increased consumption of omega-6 and omega-3 fatty acids.

The significant number of affected genes was related to signal transduction. Long-chain fatty acids can function as lipid second messengers of signaling cascade systems or as modulators and regulators of intracellular signaling pathways. The specific functions depend on the bioavailability of fatty acids (governed by their interaction with soluble proteins capable of binding fatty acids) and the lipid and protein components of biological membranes, including membrane-associated fatty acid-binding proteins [32]. In our studies, affected genes were related to membrane signaling components, such as receptors, ion channels, and membrane glycoproteins. Among them, genes belonging to several families deserve to be highlighted. The differentially expressed genes (DEG) of the ADAM family play an important role in signaling, cell adhesion, and migration. This finding was consistent with results reported by Reiss et al. [33], suggesting that free fatty acids are critical regulators of ADAM family gene function. Another family of genes is signaling membrane proteins. Our results showed that encoded by genes differentially expressed between experimental groups are G protein-coupled receptors and solute carrier proteins. Furthermore, a significant number of DEG were involved in ion transport, especially responsible for calcium and iron which showed different expression between examined groups. The regulation of the free ion concentration is not only important for signaling pathways but is also fundamental in the proper functioning of cells and the whole body. This observation confirmed the role of omega-6 and omega-3 fatty acids in ion and overall homeostasis. Likewise, affected genes were involved in the regulation of Cdc42 protein that is involved in a variety of signaling events and a number of fundamental for proper muscle functioning cellular pathways, determining cell growth, differentiation, and apoptosis. Previous genome-wide studies on pig muscle transcriptome, where compared pigs were divergent in fatty acid composition in the muscle, also pointed out the pathway dedicated to the signaling as one of the most affected [34]. The biological functions of polyunsaturated fatty acids are inter alia, due to their ability to act as secondary messengers or modulators of activities of functionally important proteins. Here, we present that adaptation to omega-6 and omega-3 supplementation is accomplished through signal transduction. Taken together, this finding indicates that the regulatory mechanisms underlying the biological effects of omega-6 and omega-3 fatty acids seem to be strictly connected to signaling pathways.

We have detected differential expression of genes related to the lipid binding between the control and experimental groups. Noteworthy are our observation of the downregulation of many apolipoprotein genes, including *APOA1*, *APOA2*, *APOA4*, *APOA5*, *APOC3*, *APOE*, and *APON*. Previous studies proved that ALA decreased the level of triacylglycerol, total cholesterol, and low-density lipoprotein (LDL) cholesterol, as well as lipoprotein-A, *APOA1*, and apolipoprotein B (*APOB*) [35]. Likewise, Fox et al. [36] perceived that the supplementation with oils containing higher content of PUFA resulted in longer term effect on decreasing the level of *APOA1* transcript in comparison to oils containing lower levels of PUFA.

Many other observations made by scientists lead to the conclusion that the control of lipoprotein metabolism through proper supplementation with bioactive components is the essential part of metabolic diseases prevention. A number of studies have shown that polyunsaturated fatty acids have a marked hypolipidemic effect. In our studies, we have detected differential expression of genes related to the lipid-binding protein between the groups of pigs. This is consistent with other research made by Canovas et al. [37], where animals showing higher levels of PUFA in the muscle were characterized by decreased level of serum lipids: total cholesterol, high-density lipoprotein (HDL) cholesterol, LDL cholesterol, and triglycerides. The diminished concentration of mentioned lipids could be related with observed in our studies downregulation of apolipoproteins.

*APOA1* and *APOA2* are the major proteins constituent of HDLs. While *APOA4* participates in intestinal lipid absorption, *APOA5* regulates VLDLs synthesis and secretion [38]. Changes in their expression may suggest regulation of this particles metabolism. Results of previous studies on the impact of PUFA on the expression of APOA family members are consistent with our findings [35, 39]. It was previously suggested that APOA level can be regulated by PUFA through molecular mechanism or indirect mechanism via changes in plasma lipid or lipoprotein concentration [40]. Nevertheless, additional studies are needed to clarify the mechanism by which omega-6 and omega-3 fatty acids affect APOA expression and further the lipoprotein metabolism.

*APOC3* is a component of chylomicrons, both very low and HDLs; inhibits the activity of lipoprotein lipase; and delays very LDLs clearance. The reduction in *APOC3* expression observed in the group supplemented with omega-3 and omega-6 fatty acid may have been a result of a direct effect of omega-3 fatty acids on a reducing *APOC3* production or as an indirect result of synthesis and secretion of fewer very LDL particles. Increased concentrations of *APOC3* are an indicator of a risk factor for coronary heart disease and atherosclerotic lesions [41]. Therefore, it could be hypothesized that reduction in *APOC3* that we observed after omega-6 and omega-3 fatty acids supplementation could have implications for coronary heart disease risk.

In accordance with our results, Canovas et al. [37] observed decreased expression of *APOE* in pigs containing increased levels of PUFA in the muscle. *APOE* removes chylomicron’s remnants from circulation through interaction with LDL receptors. It was proven that *APOE* knockout mice exhibit less body fat stores and smaller adipocytes and are more resistant to diet-induced obesity [37]. Possibly, decreased *APOE* expression that is thought to facilitate obesity decreased risk of this disorder.

It became evident that omega-6 and omega-3 fatty acids through regulation of lipoproteins play the essential role in postprandial metabolism. Therefore, they are associated with a lower risk of atherogenesis, heart disease, obesity, and diabetes. Here, we reported that omega-6 and omega-3 fatty acids affect a number of genes related to lipid transport. An important group of regulated genes belongs to apolipoprotein family. Our results highlight the need of further studies that would broaden knowledge about interactions between PUFA, lipoproteins, and metabolic disorders.

Finally, among the pathways overrepresented between both groups of animals was homeostasis. PUFA are powerful endogenous bioregulators, affecting whole body functioning and therefore homeostasis. In fact, they strongly regulate the muscle, an important organ that adjusts systemic metabolic homeostasis. First and foremost, we have observed significant changes in the expression of genes related to ion homeostasis. Homeostatic regulation of ionic gradients is critical for most functions. Here, we reported that omega-6 and omega-3 fatty acids impact expression of a number of genes involved in ion transport, including ion channels, solute carriers, G protein-coupled receptors, and tyrosine kinases. Possibly, through regulation of genes that control calcium, iron sodium and potassium ion concentration which examined fatty acids affect fundamental regulatory system of cellular processes.

Among the strengths of the present study is the scale of NGS analysis that allowed for whole genome sequencing and validation of NGS results with real-time PCR. In addition, pigs as a choice of the model organism are one of the best models for studies on effect of omega-6 and omega-3 fatty acids, which improves studies. However, limitation of lack of biological replicates should be considered in the interpretation of our results.

## Conclusions

In our study, we compared the muscle transcriptome of pigs fed with a control diet to that of pigs fed with a diet supplemented with additional fatty acids, causing different muscular fatty acid profiles. Our results showed that the omega-3 and omega-6 fatty acids regulate cellular muscle metabolism at the transcriptome level. This showed that these fatty acids are regulators of fundamental metabolic processes in muscle tissue development and functioning. A total of 749 unique genes were differently expressed between the two diet groups. The expression of 219 genes was upregulated, and the expression of 530 genes was downregulated in the group of pigs supplemented with omega-3 and omega-6 fatty acids in relation to expression in the control group pigs. The roles of these genes were indicated based on their involvement in particular biological functions. Among these biological processes were basic physiological processes such as inflammatory response, signaling pathways and lipid metabolism, leading to a finding that influence of omega-6 and omega-3 fatty on the skeletal muscle substantially impacts the whole body functioning. Obtained results reveal additional information regarding transcriptome-level regulation of biological processes underlying differences in omega-6 and omega-3 fatty acid levels.

## Additional files

Additional file 1: Heatmap of global expression levels of mRNA in the muscle of pigs from control group (M-high) and pigs fed with the diet supplemented with omega-6 and omega-3 fatty acids (M-low). The heatmap was clustered by Euclidean distance of expression. It shows gene expression in log 2 cpm (counts per million). (PDF 39 kb)
Additional file 2: List of differentially expressed genes (DEG) and results of DAVID analysis for all DEG in the porcine gluteus medius muscle tissue between pigs fed with the diet enriched for omega-6 and omega-3 fatty acids versus pigs fed with the control diet. In file are presented terms fulfilling the criteria of significance (Benjamini <0.05 and FDR <5%). (XLSX 365 kb)
Additional file 3: Additional information for the Cytoscape-ClueGo networks of differentially expressed genes (DEG) identified between pigs fed with the diet supplemented with omega-6 and omega-3 fatty acids versus pigs fed with the control diet. (XLS 46 kb)
