# Supplementary figures and images for: A porcine gluteus medius muscle genome-wide transcriptome analysis: dietary effects of omega-6 and omega-3 fatty acids on biological mechanisms
Source: Genes Nutr. 2017 Jan 31;12:4. doi: 10.1186/s12263-017-0552-8 (PMC5282897; doi:10.1186/s12263-017-0552-8)

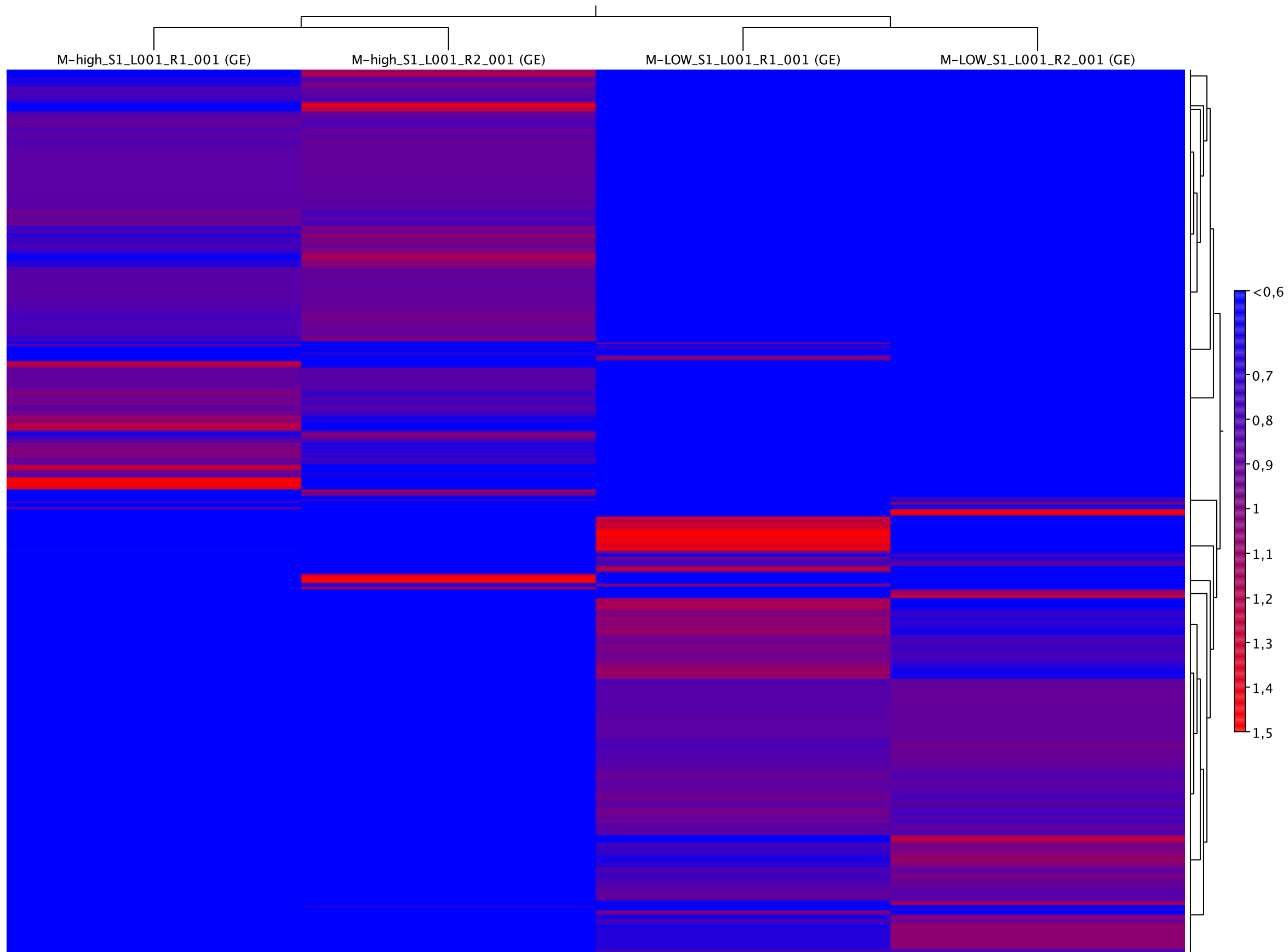

Supplement: Additional file 1: — Heatmap of global expression levels of mRNA in the muscle of pigs from control group (M-high) and pigs fed with the diet supplemented with omega-6 and omega-3 fatty acids (M-low). The heatmap was clustered by Euclidean distance of expression. It shows gene expression in log 2 cpm (counts per million). (PDF 39 kb) [file 12263_2017_552_MOESM1_ESM.pdf]
